# Book Reviews

**Published:** 1914-06

**Authors:** 


### BOOK REVIEWS

Books mentioned may be inspected at and ordered through this office. So far as possible, books received in any month will be reviewed in the issue of the second month following.

---

**The Philosophy, Poetry and Hygiene of the Kiss of Love.** Dr. J. C. Bateson, Scranton, 52 pages, $1.00, illustrated.

Our colleague has prepared a book that is certainly philosophic and poetic, by no means improper, scarcely even erotic. He has shown so much sense in discussing a sentimental subject that we are inclined to suspect that he has not had the varied and extensive experience with it which is usually demanded of an author. For amateur eugenists and sex reformers, we recommend this book as more wholesome reading than some rehashes of the Jukes and Edwards geneologies and professional parodies of what the boy who worked in the drug store used to tell the other boys when he was not professionally engaged in sweeping and running errands and washing bottles. Otherwise, we believe that the best kind of children are bred and reared by love rather than by scientific formulae and that the best antidote to immorality is genuine sentiment regarding the opposite sex. If there is any physician whose soul and sentiment have been thoroughly dessicated by the scientific atmosphere in which he lives, it may do him good to read this book.

---

**Treatment of Neurasthenia,** Dr. Paul Hartenberg, Paris translater by Ernest Playfair, M. B., M. R. C. P., Oxford University Press, American Branch, 35 W. 32, N. Y.. 282 pages, illustrated, $2.00.

For a really scientific work, this is exceptionally free from technicalities. About 50 pages are devoted to the question: What is a neurasthenic. The reader gets a good idea of what he is but the author expressly waives the ability either to formulate a definition or to explain the actual lesion. Obsessions are described as "only phobias which are past and have become chronic." Phobias are classified and described briefly but without an imposing array of Greek or other roots with phobia attached. In the treatment of sexual neurasthenia, the author is refreshingly free from hypocrisy not to say conventional ideas of morality. The book is thoroughly practical, and valuable because, throughout, the author takes the position of the medical adviser working for a humane end, rather than of a deep student, awaiting an autopsy. French being an unaccented language, it did not occur to the author but we wish it had to the translator or publisher to put an accent half an inch long over the i of neurasthenia.

---

**Treatment of Chronic Leg Ulcers, a Practical Guide to its Symptomatology, Diagnosis and Treatment.** By Dr. Edward Adams. 122 pages. Cloth $1.00. Published by The International Journal of Surgery Company, 100 William Street, New York City.

This is a trite and uninteresting subject. No brilliant and complete pathologic theory has been discovered to account for the condition. It is not one that directly threatens life. It does not belong to any particular specialist and if there is any dispute between the surgeon and the dermatologist as to which the treatment belongs, it is usually in a generous spirit of ceding it to the other. For just these reasons, we welcome this little book as one of the most valuable contributions to medical literature that has appeared in recent years. The profession has settled down to a routine treatment and a complaisantly pessimistic prognosis of one of the most disagreeable ailments which can make life miserable and for which little sympathy is elicited. The author has gone into the subject with a thorough and discriminating study of etiology and pathology as a basis for an equally discriminating therapy. He is not a hobbyist but covers the ground from ointments and lotions to resections and skin grafts.

---

**Lectures on Dietetics,** Dr. Max Einhorn, New York, published by Paul B. Hoeber, 69 E. 59, N. Y., 156 pages, illustrated, $1.00.

The edition of Diet and Nutrition having been exhausted, the author has deemed it best to publish these lectures, delivered at the N. Y. Post Graduate Medical School, in lieu of a second edition. The lectures are in Dr. Einhorn's characteristic style of delivery, lucid, convincing and occasionally witty. Chapter 1, on the principles of diet and nutrition concludes with a condensed table of principal food stuffs, giving not only content in proteid, carbohydrates and fat but also calorics. Chapter 2, on digestibility, cuts loose from the older idea of dependence on rapidity of digestion—meaning practically the time when the major part of rapidity began in the intestine—and adopts as a standard the ultimate amount of waste in the faeces. Six groups of foods are recognized, as regards digestibility, depending mainly on physical state and mechanic difficulty of extraction of nutrients. Each group accordingly, contains food stuffs not ordinarily ground together. Diet for special diseases and constitutional conditions and dietetic regimes follow, the work concluding with Dr. Einhorn's own development—duodenal feeding. While not destined to supplant larger and more detailed treatises, this book gives a good bird's eye view of the subject and it is particularly valuable in stimulating interest in a neglected subject and in dispelling a mass of crude notions that still hang about it.

---

**Arteriosclerosis**: A consideration of the prolongation of Life and Efficiency After Forty. Louis Faugeres Bishop, A. M., M. D., New York; published by the Oxford University Press, American Branch, 35 W. 32, N. Y. 383 pages, illustrated, $3.50.

This is a thorough consideration of the subject, mainly clinical but based on pathologic data and reinforced by X-ray examinations, study of polygraphic tracings, etc. A special chapter is devoted to life insurance problems. The diet is specially considered. The author has included an expression of the opinions of many clinicians both from published articles and from personal letters. The subject of blood pressure particularly with reference to compensatory high pressure and the requirements of the individual, is sensibly discussed. Cabot's investigations, showing that arteriosclerosis is not especially connected with alcoholism, either positively or negatively, are quoted. In view of the exaggerated popular conceptions of hardened arteries, and even the tendency in some professional men to employ this term to the exclusion of localized diagnosis and to reduce all practical and scientific treatise will be extensively read and pondered over.

**A Mind Remedy**, John G. Ryerson, M. D., Booton, N. J., 82 pages, price not stated.

Clinical reports are given of various diseases, such as asthma, eczema, lateral curvature, locomotor ataxia, irregular teeth, goitre, fibroids, difficult labor, leucorrhoea, puerperal convulsions and many others, have been treated with lactose. The figure, voice, mammary glands, vivacity, skin, etc., have also been improved by this treatment. The title of the book seems one that would be chosen by a sceptic rather than the author.

---

**A Hand Book for the Post-Mortem Room**, Alexander G. Gibson, D. M., F. R. C. P., Oxford. Published by the Oxford University Press, American Branch, 35 W. 32, N. Y., 140 pages, $1.50.

This is a handy and pockety compend, very concisely written and printed in small but clear type so that the amount of material contained is much greater than appears at first sight. In contrast to the prevailing American type of large and thick books, copiously and perhaps unnecessarily illustrated, a work of this scope has a competitive disadvantage. It would, however, be a poor expression of patriotism to fail to do justice to the advantages to the English type of small monographs of which this is an illustration and whose endeavor is to secure portability, to avoid unnecessary details and to facilitate reference as emergencies occur.

---

**Asthma** and its Radical Treatment, James Adam, M. A., M. D., F. R. F. P. S., Glasgow; published by Paul B. Hoeber, 69, E. 59, N. Y.; 184 pages, $1.50.

This is a clinical work, based on a sound comprehension of the pathologic and, especially, the chemic factors involved. The differentiation of such conditions as cardias and thymic asthma, the relation of asthma to eczema and urticaria, gout, etc., are well discussed. Due attention is given to hay fever and such local factors as nasal obstruction, as well as to I. Walker Hall's purin studies, without attempting to solve all problems on a single theory or to dispense with careful, personal study of the individual case.

**Biochemic Drug Assay Methods with Special Reference to the Pharmacodynamic Standardization of Drugs,** Paul S. Pittenger, Ph. G., Ph. C., Phar. D., Philadelphia, edited by F. E. Stewart, M. D., Ph. G., Philadelphia. Published by P. Blakiston's Son & Co., Philadelphia, 158 pages, 88 illustrations, $1.50.

This is a technical compend, with blank leaves for notes. The principal substances dealt with are cardiac stimulants and depressants, epinephrine, ergot, pituitary extracts, cannabis indica, with certain allied drugs and extracts. A general discussion of principles and of technical details is given. While the range of substances is somewhat narrow, the exactness with which the descriptions, even of practical points in comparative anatomy, are prepared and the excellent general discussions well prepare the student for undertaking other problems, as well as for estimating the availability of such newer methods as may be proposed.

---

**Ten Sex Talks to Girls** 14 Years and Older, I. D. Steinhardt, M. D., New York, published by the J. B. Lippincott Co., Philadelphia and London, 193 pages, 6 illustrations, $1.00.

These talks are admirably and temperately written and were originally presented as lectures. We need not discuss them in detail; they are what any physician would say, if he had the skill to put his ideas into language readily understandable by the audience addressed. The work is dedicated to "Our grandmothers, mothers, wives, sisters and daughters." At the risk of seeming old-fashioned and prudish, we feel that any work of this sort should be put into the hands of mothers, rather than of young girls, as a guide to what they themselves should impart to the latter, not all at once, but as occasion arises and also, as advice as to general conduct in which the mother should think for the daughter, rather than trust to the judgment of an immature mind and impressionable nature.

---

**A History of Laryngology and Rhinology.** By Jonathan Wright, M. D., Director of the Department of Laboratories, New York Post-Graduate Medical School and Hospital. Second Edition, Revised and Enlarged. Octave, 357 pages, illustrated. Cloth, $4.00, net. Lea & Febiger, Philadelphia and New York, 1914.

This is not only a valuable contribution to medical history but a very readable book. Among the points of interest we select: the table showing the close similarity of words for nose in the various Aryan languages; the statement of Herodotus of the high development of specialization among the Egyptians; the general notes of medical development in various ancient civilizations; the persecution of physicians on religious grounds; the detailed discussion of the history of anatomic, physiologic and therapeutic discoveries of comparatively modern or quite recent times; the excellent biographic studies. Those of us who remember when laryngology was not only an unfamiliar specialty in this part of the country but when the word was so unfamiliar that its use as a professional designation required a translation, must admit a feeling of surprise that the specialty is really so ancient. Every laryngologist should have a copy of this book and, as a matter of general medical education, every general practitioner also.

---

**Surgery; Its Principles and Practice. For Students and Practitioners.** By Astley Paston Cooper Ashhurst, A. B., M. D., F. A. C. S., Instructor in Surgery in the University of Pennsylvania; Associate Surgeon to the Episcopal Hospital; Assistant Surgeon to the Philadelphia Orthopedic Hospital and Infirmary for Nervous Diseases. Handsome large octavo, 1141 pages, with 7 colored plates and 1032 illustrations, mostly original, in the text. Cloth, $6.00, net. Lea & Febiger, Publishers, Philadelphia and New York, 1914.

It is fortunate that the name of Ashhurst, so familiar to surgeons of the last two generations, is to be perpetuated in medical literature. This work is, however, in no sense, a revised edition of that of the late John Ashhurst although due credit is given in the preface to the general information derived from the latter, but is a thoroughly modern systematic surgery. As such, it stands on its own merits and fulfills the highest modern requirements.

---

**The Junior Nurse.** By Charlotte A. Brown, R. N., Instructor in the Boston City Hospital; Graduate of the Boston City Hospital and Boston Lying-in Hospital Schools for Nurses; late Superintendent of the Hartford Hospital Training School, Hartford, Conn. 12mo, 208 pages, illustrated. Cloth, $1.50, net. Lea & Febiger, Publishers, Philadelphia and New York, 1914.

One of the best points of this work is that it discusses general hygiene, among other matters, in such a way as to enable the nurse entering a training school, to take care of herself as well as of patients. The hygiene of the feet alone is worth the price of the book. The style is simple and practical and the range of topics and degree of thoroughness with which they are discussed, is well adapted to the needs of the Junior Nurse. As long as we have "practical nurses" it would be well for physicians to recommend to them, such a work.

---

**Man's Redemption of Man—A Way of Life.** By William Osler, published by Paul B. Hoeber, 69 E. 59, N. Y., two small volumes, boxed together, $1.50.

The first of these is a lay sermon delivered at McEwan Hall, Edinburgh in 1910, the second an address to Yale students, in 1913. Both were given on Sunday and, are most appropriately of a philosophic nature, strongly tinctured with religion, but free from dogmatism. It is impossible to review these lectures in much less space than their full extent and, to endeavor to make extracts from them would, on the one hand, spoil the pleasure of the reader, on the other, give as little idea of their beauty as the exhibition of a few stones could give of the architecture of a building. As a student of medicine, we never suspected Dr. Osler of the ability which he has shown in recent years, of profundity in diagnosing and suggesting treatment for the social ills of humanity. His interests and teaching seemed rigidly limited to physical problems, along pathologic and clinical medical lines. Perhaps the pragmatism of technical scientific investigation is the best basis for philosophic consideration of broader problems. At any rate, one finds in such essays as are before us, a more penetrating quality, and one feels a deeper sense of having received benefit than in the sermons and treatises of professional moralists, sociologists and clerics.

---

**Annual Archaeologic Report,** 1913, being part of the Appendix to the Report of the Minister of Education, Ontario. This is a copiously illustrated description of some of the material in the Ontario Provincial Museum, but the Director, Rowland B. Orr, and includes the second part of a monograph by Col. Geo. E. Laidlaw on Ontario Effigy Pipes in Stone, as well as various other interesting material of the same general nature.

---

**Leben und Arbeit,** Gedanken und Erfahrungen ueber Schaffen in der Medizin, W. A. Freund, published by Julius Springer, Berlin, 170 pages, portrait of author and 10 illustrations.

We appreciate the personal inscription of this work, in English. International friendships of this sort have an important practical bearing on the development of a common, world-wide, medical science. This autobiography is not only of historic interest, but, written at a period of full maturity of experience and before senility has advanced too far—the author being only 70—it possesses much value from the practical standpoint. Three things impress us, especially: the author's original studies of congenital and developmental or, at least, non-traumatic deformities of the thorax; his insistance on the paramount value of youth, both as the period for active labor and, by association and viewpoint, for the middle aged and old man; and the candid and practical resume of the favorable and unfavorable personal factors, in regard to ultimate professional success. These are, however, mere selections from the multitude of topics well worth serious attention.

---

**Electricity in Diseases of the Eye, Ear, Nose and Throat,** W. Franklin Coleman, M. D., M. R. C. S., Chicago; published by the Courier-Herald Press. 595 pages, 156 illustrations, $5.00.

Part 1, 82 pages, is devoted to general principles of electricity and radiant energy, description of apparatus, including a very suggestive chapter on the author's own equipment. This last, though brief and modest, is of much practical value as a guide to others and it is also to be commended for giving the critic a definite basis of judgment, just as in the citation of actual conditions attending any form of experimental work. Part 2, up to page 193, treats of the general therapeutic applications of various forms of electricity and radiant energy. The remaining parts deal specifically with the diseases and abnormal conditions of the various organs mentioned. Except for the systematic arrangement and avoidance of unnecessary details, the clinical method is followed. Indeed, one might almost say the legal method, for actual evidence is presented, both by reference to personal cases, and by an elaborate study of the literature, as shown by the extensive list of authors and journal references. While the general principles of electrotherapeusis are well established, to the best of our knowledge, no similar work has appeared applying so practically and completely with this specialty or group of specialties. It is a work which gives, not dogmatic opinions or inferences, but actual experience. On account of the definiteness with which methods are described, the possible error of the personal equation is eliminated so far as possible. For the same reason, the work not only records present accomplishments, but will serve as a basis for future developments. We recommend it, therefore, not only to specialists in the lines mentioned, not only to those already particularly interested in electricity and the X-rays and other forms of radiant energy, but to all students and practitioners of medicine looking forward to perfection of such methods of treatment.

---

**Providence Retreat,** (Buffalo), 39th Report, for the year 1913.

This report, by the physician in charge, Dr. J. J. Twohey, contains an interesting summary of the work done at this excellent institution.

---

**Traitement des Stenoses Aigues du Larynx,** Dr. Guillermo Zorraquin, Chief of Clinic of Surgery at the Children's Hospital of Buenos Ayres; published by Vigot Freres, Paris. 45 pages, paper covers, 2 francs.

This work includes not only a surgical discussion of tracheotomy and intubation, but a study of the physiology and pathology of respiratory phenomena due to stenoses. Pneumograms, analogous to sphygmograms, illustrate the mechanic obstacle to free respiration, the abnormal curves showing sharp angles and a very perceptible series of interruptions, probably due to the influence of the circulatory wave, whereas the normal curve of pulmonic pressure is a slower, more gradual undulation, with less range of difference in pressure.

---

**The Road to a Healthy Old Age,** Essays, Lay and Medical, by Thomas Bodley Scott; published by Paul B. Hoeber, 69 East 59 st., N. Y. C. 104 pages, $1.00.

As indicated, part of this work is intended rather as advice to the layman of advancing years, though the latter part of the book contains a considerable amount of scientific and practical medical information, without the expected generalizations at great length on arteriosclerosis. We note, for example, the use of hippurates to relieve high arterial tension, The relief of chronic bronchitis, with and without asthma, by appropriate vaccines, consideration of thyroid failures, some very suggestive remarks as to the transiency of therapeutic agents entirely foreign to the body, as the vegetable active principles, and the emphasis laid on the choice of mineral drugs from the standpoint of foods, that is, to supply actual deficiencies in the animal chemistry.

---

**First Aid Dentistry,** E. P. R. Ryan, First Lieutenant, Dental Surgeon, U. S. A.; published by P. Plakiston's Son & Co., Philadelphia. 153 pages, 80 illustrations, $1.25.

Years ago, the reviewer happened to be situated so as to have many night calls for emergency relief of conditions which, by day, would have sought a dentist. Many physicians are so situated that their work necessarily includes attention to the teeth and the old practice of merely pulling an offending organ is unwise, while a good many conditions require something more than extraction, even as an emergency measure. Dr. Ryan's book is not written specifically for the guidance of the physician forced to usurp the function of the dentist but is rather a work for the military dentist, under conditions of emergency. Yet it serves well, the former need. From personal experience, we realize how valuable such a book would have been to the young physician and, considering the circle of readers which we reach, it may be pardonable to emphasize this function of the book rather than its special usefulness to the dentists, civil or military.

---

**Modern Surgery: General and Operative,** by J. Chalmers DaCosta, M. D., Samuel D. Gross, Professor of Surgery, Jefferson Medical College, Philadelphia, Pa. Seventh Edition, Revised, Enlarged and Reset. Octavo of 1515 pages, with 1085 illustrations, some of them in colors. Philadelphia and London: W. B. Saunders Company, 1914. Cloth, $6.00, net; Half Morocco, $7.50, net.

It is scarcely necessary to review at length, a work so well known in its earlier editions. We are impressed with the fact that this work is not merely a description of operative technic but a treatise on surgery. The first quarter of the book is devoted to bacteriology, surgical pathology and the diseases commonly considered as essentially surgical, such as erysipelas, tetanus, syphilis, etc. The very practical discussion of antiseptic chemicals is especially to be commended. The philosophy of surgical disease is discussed so as to afford a good conception of causes, methods of prophylaxis and treatment, as well as of pathology. Anaesthesia, bandaging, skiagraphy, injuries by electricity, methods of controlling pain and inflammation, are among the subjects carefully and thoroughly treated, and showing that the author has taken a broad and deep view of surgery.

---

**Clinical Hematology: An Introduction to the Clinical Study of the so-called Blood Diseases and of Allied Disorders,** by Gordon R. Ward, M. D.; Fellow of the Royal Society of Medicine, Medical Society of London, etc. Octavo of 394 pages, illustrated. Philadelphia and London: W. B. Saunders Company, 1914. Cloth, $3.50, net.

A work on haematology without a colored plate of blood cells and malarial plasmodia, is a surprise in these days and while such charts are contained in other works or distributed as advertisements, and sometimes raise false hopes of what the inexpert haematologist may accomplish, we are rather dubious as to the wisdom of omitting them entirely. The cuts of red and white cells are scarcely adequate. In other words, the publishers in presenting an English work, have not fulfilled the American demand for copious and elaborate illustrations which they themselves have done as much as anyone to create. Considered, not as a picture book, but as one for serious reading, this is a work deserving careful and general study. The author lists, classifies, and describes 40 diseases. Cholaemia, haemophilia, purpura, cyanosis, methaemogloninaemia, intravascular cyto-phagocytosis are among the conditions not always included in works on haematology. The blood forming organs and the remote results of their lesions and disturbances, are thoroughly treated. In the chapter on the Blood in Various Diseases, a considerable amount of information as to leucocytes, secondary anaemia, effects of parasites and bacteria, etc., is brought together in systematic order. The book is not merely one on clinical diagnosis or even pathology, but, in addition, it is thoroughly clinical and pays great attention to therapeutics.

---

**Psychanalysis: Its Theories and Practical Application,** by A. A. Brill, Ph. B., M. D., Chief of Clinic of Psychiatry and Clinical Assistant in Neurology, Columbia University Medical School; Chief of the Neurological Department of the Bronx Hospital and Dispensary. Second Edition, thoroughly revised. Octavo of 393 pages. Philadelphia and London: W. B. Saunders Company, 1914. Cloth, $3.00, net.

To one accustomed to deal rather with organic disease than functional disturbances and with functional disturbances rather as expressions of concealed lesions or as conditions having only a slight relation to the conscious mind, the detailed discussion of history and subjective experiences, seems at first, somewhat exaggerated. It should be remembered, however, that there is a vast field of functional nervous disorders and of disturbances of mentality far short of insanity, which deserve the careful and minute analysis admittedly due to chemic and mechanic disturbances of visceral physiology and to the results of demonstrable lesions. Sex plays a large part in human thought. Whether it is so universally dominant as this book assumes, in interpreting dreams, imperative notions, habits of thought, etc., seems to us debatable. But, whatever one's own opinions may be, it must be admitted that there is a considerable number of unfortunate individuals, not sufficiently deranged to be confined in insane asylums, not criminal to the extent of being incarcerated, whose mental life is filthy, cruel, silly or otherwise abnormal. Many of these individuals are near the line the crossing of which means danger to others; many are conscientious enough to be constantly tortured by a realization of their own morbidity and are hampered by the diversion of mental energy into the repression of indecent and foolish impulses, which might otherwise be put to good advantage. The careful analysis of the psychology of such individuals, in many instances, suggests extrinsic causes that may be removed; in others, it shows the origin of an obsession in some trivial impression, perhaps dating back to childhood and the mere discovery of the origin and subzsequent trend of abnormal thoughts, may help the patient who is sincere in his wish to be rid of them.

---

**Infant Feeding**, by Clifford G. Grulee, A. M., M. D., Assistant Professor of Pediatrics at Rush Medical College, Chief of Pediatric Staff, Cook County Hospital. Second Edition, thoroughly revised. Octavo of 314 pages, illustrated. Philadelphia and London: W. B. Saunders Company, 1914. Cloth, $3.00, net.

That this book has come to a second edition within a year and a half, is an indication of its merit, and of the professional appreciation which we anticipated at the time of the review of the first edition. As the author states in the preface, there has been no revolutionary change of ideas of dietetics, but a number of advances have been made, which have been incorporated in the present edition. The work is systematic, comprehensive, practical, and the author is sensible and free from hobbies.

